# Pressure induced metallization with absence of structural transition in layered molybdenum diselenide

**DOI:** 10.1038/ncomms8312

**Published:** 2015-06-19

**Authors:** Zhao Zhao, Haijun Zhang, Hongtao Yuan, Shibing Wang, Yu Lin, Qiaoshi Zeng, Gang Xu, Zhenxian Liu, G. K. Solanki, K. D. Patel, Yi Cui, Harold Y. Hwang, Wendy L. Mao

**Affiliations:** 1 Department of Physics, Stanford University, Stanford, CA 94305, USA; 2 National Laboratory of Solid State Microstructures and Department of Physics, Nanjing University, Nanjing 210093 China; 3 Geballe Laboratory for Advanced Materials, Stanford University, Stanford, CA 94305, USA; 4 Stanford Institute for Materials and Energy Sciences, SLAC National Accelerator Laboratory, Menlo Park, CA 94025, USA; 5 Department of Geological Sciences, Stanford University, Stanford, CA 94305, USA; 6 HPSynC, Geophysical Laboratory, Carnegie Institution of Washington, Argonne, IL 60439, USA; 7 Center for High Pressure Science and Technology Advanced Research, Shanghai 201203, China; 8 Geophysical Laboratory, Carnegie Institution of Washington, Washington, DC 20015, USA; 9 Department of Physics, Sardar Patel University, Vallabh Vidyanagar, Gujarat 388120, India

## Abstract

Layered transition-metal dichalcogenides have emerged as exciting material systems with atomically thin geometries and unique electronic properties. Pressure is a powerful tool for continuously tuning their crystal and electronic structures away from the pristine states. Here, we systematically investigated the pressurized behavior of MoSe_2_ up to ∼60 GPa using multiple experimental techniques and *ab-initio* calculations. MoSe_2_ evolves from an anisotropic two-dimensional layered network to a three-dimensional structure without a structural transition, which is a complete contrast to MoS_2_. The role of the chalcogenide anions in stabilizing different layered patterns is underscored by our layer sliding calculations. MoSe_2_ possesses highly tunable transport properties under pressure, determined by the gradual narrowing of its band-gap followed by metallization. The continuous tuning of its electronic structure and band-gap in the range of visible light to infrared suggest possible energy-variable optoelectronics applications in pressurized transition-metal dichalcogenides.

## Abstract (web summary)

Controlling the properties of transition metal chalcogenides enables the study of new condensed-matter phenomena and the development of potential applications. Here, the authors continuously tune the crystal and electronic structure of molybdenum diselenide away from the pristine state using pressure.

## Graphical abstract

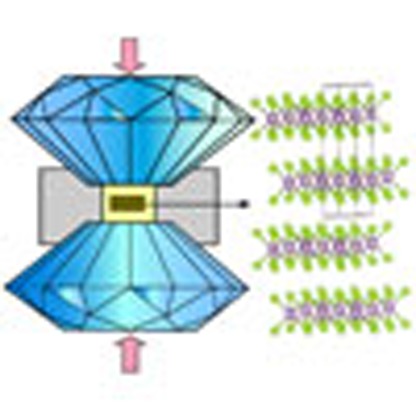

Transition-metal dichalcogenides (TMDs) 2H-MX_2_ (M=Mo, W, and etc, X=S, Se, and Te) have recently attracted intense scientific and engineering interest because of their ease of fabrication and unique electronic structure^1^,^2^,^3^,^4^,^5^,^6^,^7^,^8^,^9^,^10^,^11^,^12^,^13^,^14^,^15^,^16^. TMDs have strong chemical bonding within each X-M-X trilayer and weak van der Waals (vdW) interaction between neighbor trilayers in their crystal structures. This quasi two-dimensional (2D) nature grants TMDs facile three-dimensional (3D) to 2D crossover through methods like exfoliation^1^,^2^,^4^,^5^,^8^,^9^,^12^. Band structure engineering on TMDs allows ones to explore exotic condensed matter phenomena and develop many potential applications. For example, the modification of their band structures from indirect-band-gap to direct-band-gap provides insights into opto-electronics and valley electronics^11^,^14^,^15^,^16^,^17^,^18^. So far, the electronic structure engineering of TMDs has mainly been achieved through the following experimental methods: (i) applying electrical field to control the spin splitting and freedom of electrons^14^,^16^, (ii) utilizing quantum confinement with samples thinning down into monolayers of MX_2_^11^,^15^,^17^,^18^, and (iii) employing stress or strain (by bending or stretching the thin films or employing substrates with different lattice constants)^19^,^20^, as suggested by calculations^18^,^21^,^22^,^23^,^24^,^25^,^26^.

Compared with the three methods mentioned above, high pressure is a powerful way to induce dramatic changes in their crystal structures and electronic structures^27^,^28^. This qualifies high pressure as a desirable approach to explore the tunability of TMDs. In particular, the ability to continuously tune the crystal and electronic structures away from the pristine states is crucial to a wide array of applications, e.g., electromechanical devices, energy-variable opto-electronics, and energy-variable photovoltaics. Various pressure-induced electronic evolutions such as semiconductor to metal transitions have been observed in different materials^29^,^30^,^31^. However, many of these electronic transitions were accompanied by first-order structural transitions. A pressure-induced first-order structural transition, by definition, involves a discontinuous change in the volume (of the unit cell). And the corresponding discontinuity in its electronic structure could limit the energy tunability for potential opto-electronic or photovoltaic applications. To overcome this challenge, we need to discover TMDs with continuous structural and electronic response.

Previous high pressure studies on MoS_2_ clearly demonstrated that a first-order structural transition (2H_c_-MoS_2_ to 2H_a_-MoS_2_) occurred close to metallization^32^,^33^,^34^,^35^,^36^. This transition may relate to the vdW interactions in between neighbor S-Mo-S trilayers^36^. To prevent the 2H_c_ to 2H_a_ transition, the substitution of chalcogenide anions in MoS_2_ is a potential route. Because Se^2−^ has broader electron orbitals than S^2−^'s that lead to stronger interlayer interactions, MoSe_2_ may have totally different high pressure behavior. Experimentally, the structural and electronic behavior of compressed MoSe_2_ remains to be fully explored^37^,^38^,^39^. Interestingly, recent calculations have predicted that MoSe_2_ metallizes between 28 to 40 GPa while preserving the 2H_c_ structure^40^.

In our work, high pressure up to ∼60 GPa was generated by a diamond anvil cell (DAC). X-ray diffraction (XRD) data and Raman spectra data indicate that MoSe_2_ preserves the 2H_c_ structure without any structural transition. *Ab-initio* calculations for modeling the layer sliding process are presented to understand the contrasting behavior between MoSe_2_ and MoS_2_, and further predict the structural stability of MoTe_2_. Infrared (IR) spectra data and temperature-dependent electrical resistivity data demonstrate the highly tunable transport properties of MoSe_2_. Electronically, both experiments and calculations show that pressure strongly modulates its band structure from semiconducting to metallic.

## Results

### XRD and Raman spectroscopy

**Figure 1:**
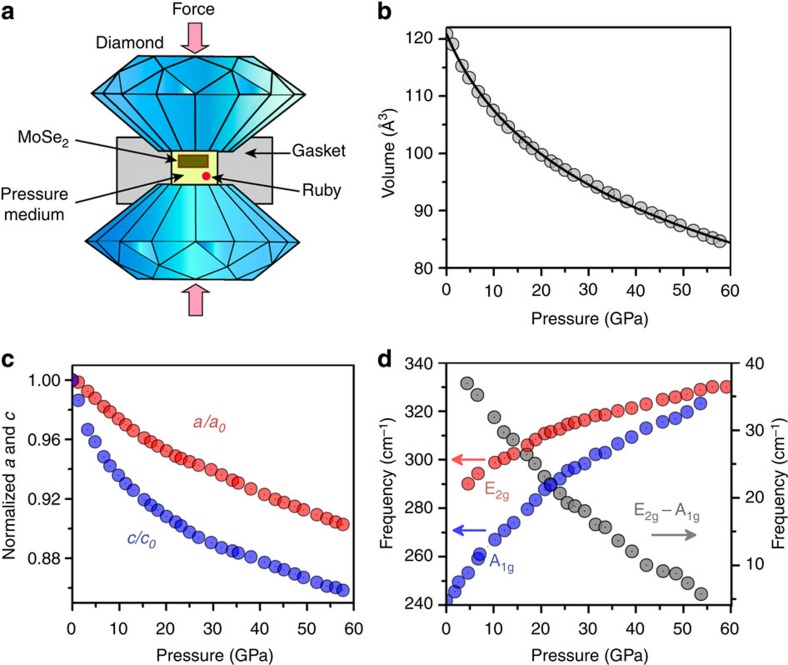
Experimental set up and structural and vibrational responses under pressure. (**a**) Schematic of the high pressure DAC set up. (**b**) Pressure-volume data from XRD measurement and the curve represents a third-order BM-EOS fitting. (**c**) Normalized cell parameters *a*/*a*_0_ and *c*/*c*_0_ versus pressure. The error bars given by EXPGUI-GSAS are smaller than the size of the markers. (**d**) Evolution of vibrational modes A_1g_ and E_2g_ and their difference (E_2g_−A_1g_) under pressure, measured by Raman spectroscopy.

The experimental set-up of the DAC is shown in Fig. 1a. Under compression, all XRD peaks for MoSe_2_ continuously shift to larger 2*θ* (smaller *d*-spacing) and no new peak is observed (Supplementary Fig. 1 and Supplementary Note 1). Decompression of the sample shows the shifts of all peaks are reversible. All patterns are consistent with the 2H_c_ structure and representative Rietveld refinements are shown in Supplementary Fig. 2 and Supplementary Table 1. The absence of a first-order structural transition is further supported by equation of state (EOS) data in Fig. 1b and normalized cell parameters versus pressure data in Fig. 1c, as neither of them exhibits any discontinuity. To fit the pressure-volume relation, a third-order Birch-Murnaghan (BM) EOS is employed. With unit-cell volume *V*_0_ fixed at the experimental value of 120.8 Å^3^, the fitting yields a bulk modulus *B*_0_=62(1) GPa and a derivative of bulk modulus *B*'=5.6(1). The relatively large value of *B*' suggests a strong change of volume compressibility under pressure.

**Figure 2:**
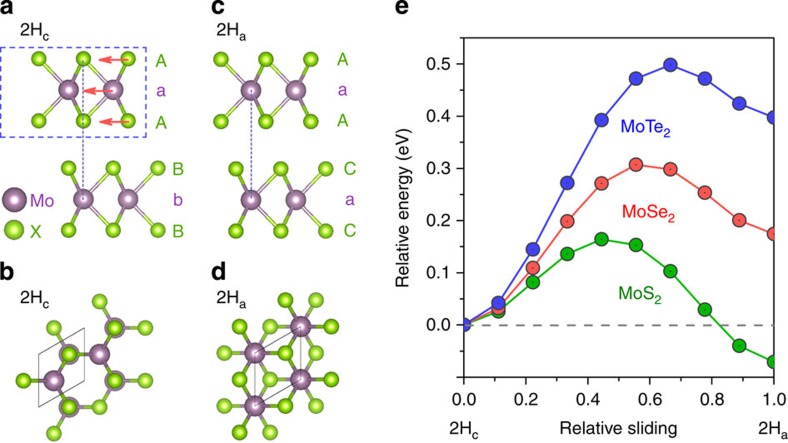
Structural details of 2H_c_− and 2H_a_−MoX_2_ and layer sliding calculations. (**a**) The side view (projected on *ac* plane) of 2H_c_ structure in MoS_2_, MoSe_2_ and MoTe_2_. X represents S, Se and Te. The red arrows represent one sliding path for the 2H_c_ to 2H_a_ transition, where one unit of X-Mo-X triple layers (marked by a blue box) shifts systematically in *ab* plane. (**b**) The top view (projected on *ab* plane) of 2H_c_ structure. (**d**) The side view of 2H_a_ structure. (**c**) The top view of 2H_a_ structure. (**e**) The total energy of MoS_2_, MoSe_2_ and MoTe_2_ as a function of relative sliding from 2H_c_ to 2H_a_. The total energies of 2H_c_ structures are set to be zero as references, marked by a broken line. For MoS_2_, the unit cell volume was fixed at experimental data under 20 GPa^35^. After electronic relaxation of the atomic positions, the S-Mo-S layer distance of structure was set to be a constant during the layer sliding. The same procedures were performed on MoSe_2_ under 23 GPa (our experimental data) and MoTe_2_ under 20 GPa (theoretical data^40^).

At ambient conditions, the structure of 2H_c_-MoSe_2_ features the X-M-X triple layers linked via vdW forces (Fig. 2a,b)^41^,^42^. During the initial compression, cell parameter *c* is much more compressible than *a* due to the weak vdW interactions in between Se-Se layers along *c* (Fig. 1c), while higher pressure allows them to have nearly isotropic contractions. At the highest pressure ∼60 GPa, *a* and *c* reduce by ∼10 and ∼15% respectively. The gradual closure of the vdW gap is suggested by tracking the ratio of Se-Mo layer distance to Se-Se layer distance, where it drops fast at low pressure but decreases much slower at high pressure (Supplementary Fig. 3). In addition, we measured the Raman spectra under pressure (Supplementary Fig. 4 and Supplementary Note 2). See Fig. 1d, the vibrational modes A_1g_ and E_2g_, and the spacing between them shift successively under pressure. These observations indicate that the crystal structure of MoSe_2_ continuously evolves from a quasi 2D structure to an isotropic 3D one without a structural transition.

### Structural calculations

Our XRD and Raman results appear to be surprising—At ambient conditions, MoS_2_ and MoSe_2_ are iso-structural in crystal structures and possess highly similar electronic structures, and it is therefore natural to assume that the 2H_c_ to 2H_a_ transition^32^,^33^,^34^,^35^ would also occur in MoSe_2_. Nonetheless, 2H_a_ structure does not fit the XRD patterns of MoSe_2_ in the entire pressure region studied in this work. Though bearing highly similar Mo-Se chemical environments and the same space group (*P*6_3_/mmc), 2H_a_ structure and 2H_c_ structure have distinct structural topologies. In 2H_a_ structure Mo occupies a 2b Wyckoff position while in 2H_c_ structure it occupies a 2c Wyckoff position. Also, the two adjacent units of X-Mo-X triple layers have contrasting stacking patterns in 2H_c_ and 2H_a_ (shown in Fig. 2a–d). To seek theoretical support of the structural stability of 2H_c_-MoSe_2_, we performed two sets of *ab-initio* calculations. We first confirmed that 2H_c_-MoSe_2_ is more stable than 2H_a_-MoSe_2_, based on the experimental unit cell at the highest pressure ∼60 GPa. This is consistent with recent calculations showing the enthalpy difference between 2H_a_ and 2H_c_ of MoSe_2_ keeps increasing from ambient pressure up to at least 130 GPa^40^. We then calculated the cell parameters at different volumes based on 2H_c_-MoSe_2_ and the results agree well with our experimental data (Supplementary Fig. 5 and Supplementary Note 3). Note that the small discrepancy at low pressure comes from the limitations of *ab-initio* calculations in describing the vdW interaction.

In order to understand the contrasting structural behavior between MoS_2_^32^,^33^,^34^,^35^ and MoSe_2_^40^, and see whether there is a predictable trend in TMDs, we further carried out layer sliding simulations for MoS_2_, MoSe_2_, and MoTe_2_ at ∼20 GPa. The side and top views of 2H_c_-type and 2H_a_-type structures are shown in Fig. 2a–d. The transition from 2H_c_ to 2H_a_ can be realized by systematically shifting half of the atoms (one unit of X-Mo-X triple layers) in a unit cell. As illustrated in Fig. 2a, we defined one sliding path by the red arrows for this transition. For MoS_2_, the unit-cell volume is fixed at the experimental value at 20 GPa^35^. After initial relaxation of the atomic positions within the 2H_c_-type structure, the S-Mo-S layer distances are fixed during the layer sliding. The same procedures are performed for MoSe_2_ at 23 GPa (using our experimental data) and MoTe_2_ at 20 GPa (based on previous theoretical data^40^). The total energies of 2H_c_ are set to be zero as the references. Fig. 2e shows the relative energies as a function of the relative layer sliding, i.e., 0 represents 2H_c_ and 1 represents 2H_a_. MoX_2_ needs to overcome an energy barrier in order to undergo the 2H_c_ to 2H_a_ transition. The maximum energy barrier is ∼0.3 eV for MoSe_2_ and ∼0.5 eV for MoTe_2_, which are significantly higher than ∼0.15 eV for MoS_2_. More importantly, 2H_a_-MoSe_2_ and 2H_a_-MoTe_2_ bear higher energies than the initial 2H_c_ structures, which would make this transition unfavorable. However, in the case of MoS_2_, the 2H_a_ structure becomes energetically favorable. Note that the X-Mo-X layer distance is fixed in this set of calculations. Realistically in compressed MoS_2_, the S-Mo-S distance and unit cell volume drop during the 2H_c_ to 2H_a_ transition^32^,^33^,^34^,^35^, which allows the total change in enthalpy to be continuous at zero temperature.

### IR spectroscopy

**Figure 3:**
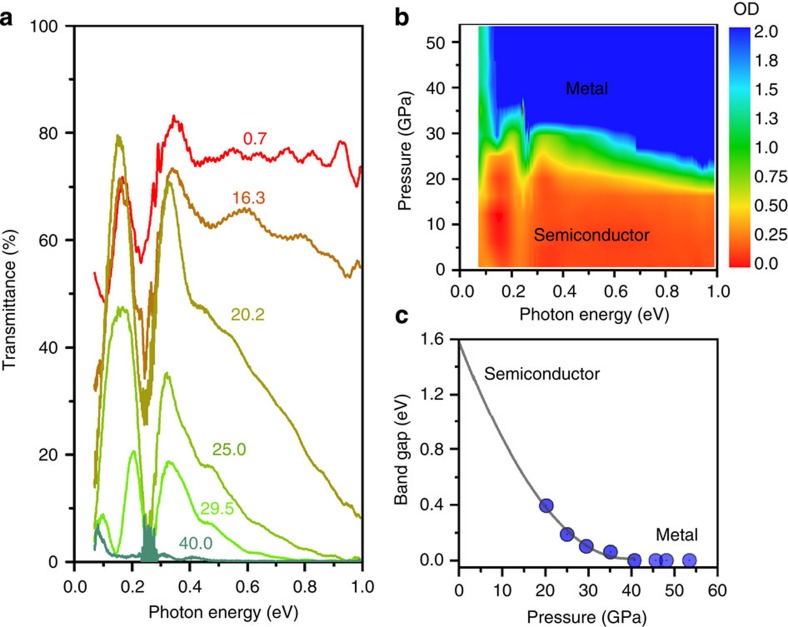
IR transmittance spectra and analysis. (**a**) Representative IR transmittance spectra at high pressures, numbers show pressures in unit of GPa. The 0.23–0.28 eV region is obscured by diamond phonon absorptions from the DAC. (**b**) Pressure−energy−optical density (OD) contour, OD is defined as −log(*T*) while *T* is the transmittance. (**c**) Evolution of band gap under pressure. Circles are extrapolated indirect band gaps and the curve shows a parabolic fit of the band gap versus pressure. The band gap closure is observed at ∼40 GPa.

The lattice response of MoSe_2_ at high pressure will inevitably change its electronic structure, and thus its optical properties which strongly depend on the electronic structure. Our data shows that MoSe_2_ undergoes a large electronic evolution where band-gap narrowing is followed by metallization of MoSe_2_. Fig. 3 shows the measured synchrotron IR spectra and its analysis (details are shown in Supplementary Note 4). Below 16.3 GPa, the transmittance spectra (Fig. 3a) look similar, where a transmission window extends from 0.06 to 1.0 eV. At pressure above 20.2 GPa, the 0.3–1.0 eV region develops into a tilted curve and keeps collapsing into lower energy region, indicating the band-gap's narrowing. At above 40.7 GPa to the highest pressure, nearly zero transmission is observed in between 0.15 to 1.0 eV, suggesting the metallization of MoSe_2_. Another way to interpret the IR data is from the viewpoint of the optical density (OD) *A*_*λ*_ (see Supplementary Fig. 6 for the plot of OD versus energy). OD or *A*_*λ*_ is defined as −log*T* (*T* as the transmittance). It can be easily seen from the energy-pressure-OD map in Fig. 3b that a clear changeover of low OD (in semiconducting state) to high OD (in metallic state) occurs between 20 to 35 GPa.

For an indirect-band-gap semiconductor, the absorption coefficient is proportional to the square of the energy difference of the photon energy and band-gap^43^. Using this empirical model for semiconductors, we obtained the indirect-band-gap *E*_g_ via linear extrapolations of (*hνA*_*λ*_)^1/2^ where *hν* is the photon energy. A representative extrapolation is shown in Supplementary Fig. 7. The fitted *E*_g_ value at 20.2 GPa is 0.4 eV. From 20.2 to 35.1 GPa, *E*_g_ keeps decreasing (see Fig. 3c). From 40.7 GPa to the highest pressure, *E*_g_ is nominally zero. We notice that the trend of band-gap decrease at below 41 GPa could not be well described by a linear fitting. The non-linearity is also shown in previous calculations on band-gap's dependence on applied strain^23^. The lack of data points and inaccuracies in optical measurement prevent us from determining the best function for the band-gap−pressure relation. However, as a simple approach to guide eyes, we fit the data with a parabolic curve, which yields *E*_*g*_=−0.08(2) *P*+0.0010(3) *P*^*2*^, indirect-band-gap (*E*_g_) in unit of eV and pressure (*P*) in unit of GPa. The extrapolated band-gap at ambient pressure is 1.6(3) eV, which is in good agreement with previous reports^18^,^44^,^45^.

### Electrical resistivity

**Figure 4:**
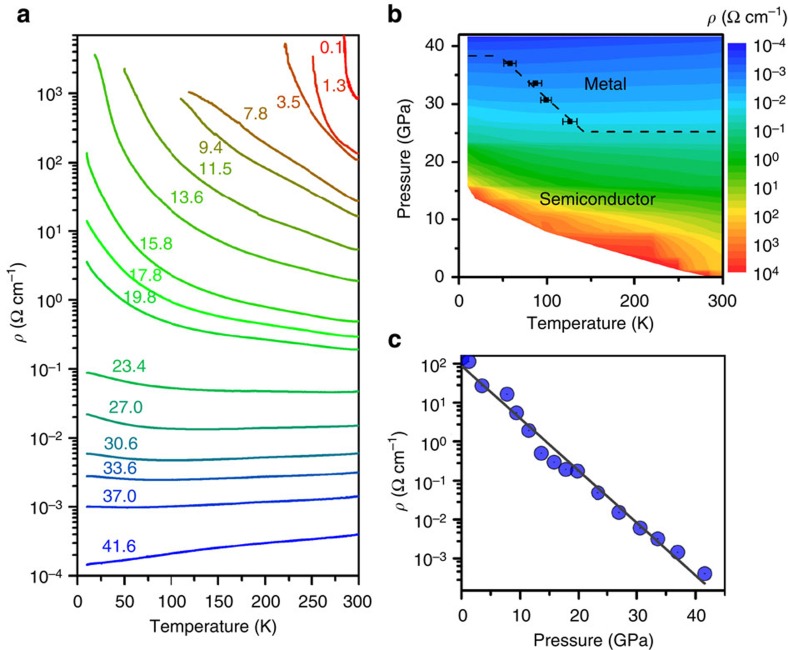
Temperature-dependent resistivity data and analysis. (**a**) Temperature-resistivity curves at different pressure, numbers show pressures in GPa. (**b**) Temperature-pressure-resistivity contour map. (**c**) Room temperature resistivity versus pressure, the line shows a linear fitting of log ρ versus pressure (equivalent for an exponential fitting of ρ versus pressure).

We also measured the temperature-dependent resistivity up to ∼42 GPa (Supplementary Note 5 and Supplementary Fig. 8). At low pressures (Fig. 4a), the temperature (*T*)−resistivity (ρ) curves at below 23.4 GPa exhibit negative *dρ/dT* throughout all temperatures, indicating the presence of a semiconducting state. From 27.0 to 37.0 GPa, the high-temperature region shows positive *dρ/dT* whereas the low-temperature region has negative *dρ/dT* (see Fig. 4b). At above 41.0 GPa, positive *dρ/dT* is observed in all temperatures, implying the metallization of MoSe_2_. A comparison of our room temperature resistivity data on MoSe_2_ (Fig. 4c) with previous data on MoS_2_ shows that there are dissimilar trends in between them^34^,^35^. For MoS_2_, the room temperature resistivity dropped strongly at below ∼15 GPa and then reached a plateau at higher pressure^34^,^35^, which was related to a first-order structural transition. In sharp contrast, for MoSe_2_, the decrease of its room temperature resistivity is nearly exponential, fit by log (*ρ*)=1.9(1)—0.134(5) *P*, resistivity (ρ) in unit of Ω cm^−1^ and pressure (P) in unit of GPa. Pressure allows the room temperature electrical resistivity of MoSe_2_ to decrease more than six orders of magnitude from ambient to 41.6 GPa.

### Electronic structure

**Figure 5:**
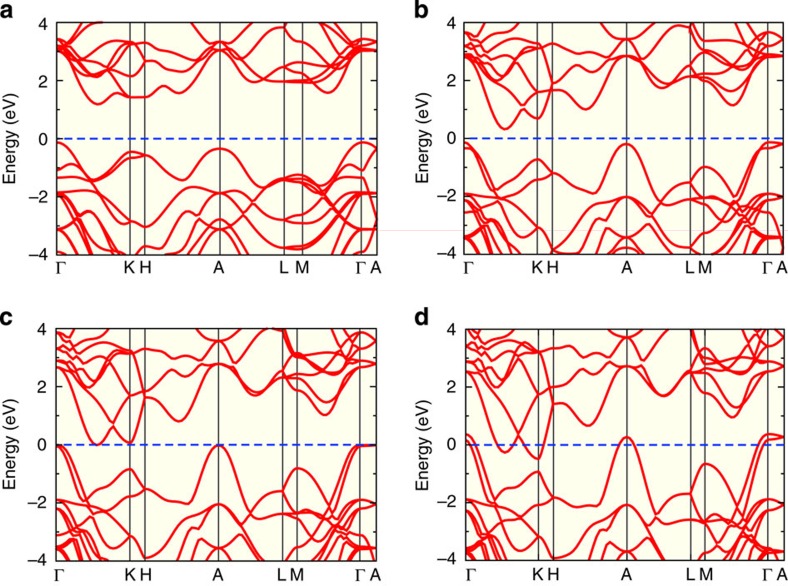
Calculated band structures of *2H*_*c*_-MoSe_2_. (**a**) Ambient pressure, (**b**) 23 GPa, (**c**) 41 GPa, and (**d**) 58 GPa. Blue dotted line shows the Fermi level (*E*_F_).

To better understand the electronic evolution of MoSe_2_ that determines its highly tunable optical and electrical transport properties, we performed *ab-initio* calculations on the electronic structure of MoSe_2_ at four representative pressures. The results undoubtedly demonstrate the pressure-induced band-gap narrowing and metallization. At ambient pressure, seen from Fig. 5a, the band structure is consistent with previous results^18^,^44^,^45^. It has a direct-band-gap *E*_K-K_ (∼1.8 eV) at K and an indirect-band-gap (∼1.3 eV) that locates in between Γ and Γ-K conduction band (CB) valley. The bottom of CB between Γ and K mainly origins from the Mo *dxy* and *dx*^*2*^−*y*^*2*^ orbitals, and the top of valence bands (VBs) at Γ comes from the Mo *dz*^*2*^ orbital. Meanwhile, the *dxz* and *dyz* dominated CBs are further above from the Fermi-level (*E*_F_). Higher pressure results in a strong decrease of its indirect-band-gap and induces large movements of the orbitals towards the *E*_*F*_. At 23 GPa, shown in Fig. 5b, the indirect-band-gap becomes as small as 0.5 eV. Albert decreasing, the direct-band-gaps remain at large values, e.g., *E*_K-K_ is ∼1.4 eV. Remarkably, pressure allows the *dxz* and *dyz* orbitals to gain more overlap with Se *p* orbitals and thus largely widen their band dispersions. In comparison, the *dxy* and *dx*^*2*^*−y*^*2*^ orbitals are less impacted due to smaller overlap with Se *p* orbitals. As a consequence (see Supplementary Note 6), one *dxz* and *dyz* dominated CB quickly goes down at K point and forms two CB valley minimum together with the previous *dxy* and *dx*^*2*^−*y*^*2*^ dominated CB.

Metallic band structures are obtained by further increasing the pressure. For example, seen from Fig. 5c at 41 GPa, there lies density of states across the *E*_F_. The *dxy* and *dx*^*2*^−*y*^*2*^ dominated CB valley minimum crosses below the *E*_F_, while the other CB valley minimum is still slightly above the *E*_F_ (see Supplementary Fig. 9). At 58 GPa, shown by Fig. 5d, both CB valley minima cross below the *E*_F_. It is worth mentioning that even at as high as 58 GPa, the ‘indirect' feature of the band structure is still well maintained: although the CBs and VBs overlap in energy range, no direct cross is seen. To be specific, the energy separation at K is as large as ∼0.6 eV. Meanwhile, the relative shifts of CBs and VBs generate a number of hole pockets (e.g., at Γ and A) and electron pockets (e.g., at K). These pockets may largely affect the low-temperature electrical and optical transport properties of MoSe_2_.

## Discussions

Our experiments and calculations clearly demonstrate the absence of structural transition in MoSe_2_. One empirical understanding of the contrasting behavior in MoS_2_^32^,^33^,^34^,^35^,^36^ and MoSe_2_ involves the different localizations of *p* orbitals among chalcogenide anions S^2−^, Se^2−^, and Te^2−^. The 3*p* orbitals of S^2−^ dominate the electronic structure in MoS_2_ while the 4*p* and 5*p* orbitals are primary for MoSe_2_ and MoTe_2_ correspondingly. 4*p* and 5*p* orbitals are much more delocalized than 3*p* orbitals, which would give rise to strong interaction within the vdW gaps of MoSe_2_ and MoTe_2_ to prevent this sliding process, vice versa for MoS_2_. We can safely conclude that it is easier for 2H_c_-MoS_2_ to experience a structural transition through sliding in between neighbor S-Mo-S layers, but this does not apply to MoSe_2_ or MoTe_2_. Beside from the effects of chalcogenides anions, the effects of transition metal cation should also be considered in determining the stabilities of layered structures. For example, recent calculations proposed that the interlayer Mo-Mo *d*-electron propagation should be considered in determining the stability of layered structures^40^. More importantly, size effect of different transition metal cations is also expected to change the interlayer interactions. Previous studies on WS_2_ and WSe_2_^46^,^47^,^48^ showed that they experience continuous lattice contractions under pressure. W^2+^ has broader electron orbitals and may introduce stronger interlayer interactions than Mo^2+^, which results in the absence of layer sliding in WS_2_. In a more general perspective, stronger interlayer interactions help stabilize the structures of TMDs and are more likely to yield continuous lattice response under extreme environments such as pressure.

Previous studies have reported many electronic transitions such as insulator to metal or semiconductor to metal transitions in the group of binary chalcogenides, see for example, Bi_2_X_3_^31^,^49^,^50^, Sb_2_X_3_^51^,^52^,^53^, and Ag_2_X^54^,^55^,^56^. For structures starting with vdW gaps at ambient conditions, the closure of their vdW gaps is generally accompanied or followed by first-order structural transitions where large structural re-constructions or atomic movements take place^43^,^44^,^45^,^46^,^47^,^48^. However, in the case of MoSe_2_, the metallization process does not involve any sudden change in the crystal structure, which allows its electronic structure to be continuously tuned. Through multiple experimental techniques combined with *ab-initio* calculations, we demonstrate that the band-gap of MoSe_2_ (in the range of visible to IR region) exhibits a strong dependence on pressure. This may allow MoSe_2_, one representative TMD, to be applied in energy-variable opto-electronics and photovoltaics, although the limitation of sample size (0.05 to 0.1 mm) must be taken into account in future investigation.

Compared with others methods of band structure engineering approachable by experiments^11^,^12^,^13^,^14^,^15^,^16^,^17^,^18^, pressure is the only way to metalize MoSe_2_ and MoS_2_^34^,^35^. This highlights pressure's dramatic role in tuning the electronic properties of TMDs. Our different aspects of the experimental data show good agreement with the recent calculations on MoSe_2_^40^. This further suggests 2H_c_-MoSe_2_ a suitable material with a concise unit cell for testing and improving first-principles calculations that can be probed by experiments. If future experiments, i.e., by applying non-uniaxial compression, are able break the inversion symmetry of the crystal structure of pressurized MoSe_2_, the spin and valley electronics of MoSe_2_ would be largely different when we consider the large shifts of the CBs and VBs^5^,^12^,^14^,^16^. More importantly, the novel scenario of excitonic insulator may be experimentally realizable in MoSe_2_^40^, while in MoS_2_ the complexity of the layer sliding structural transition may prevent the formation of this electronic state^32^,^33^,^34^,^35^,^36^. For the pressurized metallic MoSe_2_, its distinguished ‘indirect' band structures and electronics state filled with electron holes and pockets await further exploitation in condensed matters physics, i.e., charge density wave or superconductivity may be found in TMDs at higher pressure^35^,^36^,^40^.

In conclusion, we comprehensively studied the high pressure behavior of MoSe_2_ up to ∼60 GP through a series of structural, vibrational, optical, and electrical measurements combined with *ab-initio* calculations. 2H_c_-MoSe_2_ evolves from an anisotropic 2D layered structure to an isotropic 3D one without any sudden structural change under pressure. Our layer sliding calculations highlight the role of the chalcogenide anions in stabilizing either 2H_a_ or 2H_c_ layered patterns. Electronically, MoSe_2_ undergoes a semiconductor to metal transition, and correspondingly exhibits highly tunable optical and electrical properties. Upon compression, the ‘indirect' feature of its electronic structure is robustly conserved with the appearance of two conduction band minima. The large and continuous tuning of its electronic structure may have potential applications in energy-variable (visible to IR) opto-electronics and photovoltaics.

## Methods

### Sample growth

High quality stoichiometric MoSe_2_ single crystals were grown by direct vapor transport technique^39^,^44^,^57^. Elemental Mo and Se (99.9% purity, purchased from *Koch Light Ltd.*) of the stoichiometric ratio were sealed in a quartz ampoule at pressure better than 10^−5^ Torr. The ampoule was placed in a two-zone horizontal furnace where the temperatures were slowly raised from room temperature to 1,060 and 1,080 °C for growth zone and source zone respectively. This temperature gradient was then maintained for ∼188 h to produce to single crystal platelets of MoSe_2_. The shiny and gray crystals have a typical thickness of ∼4 μm and area of ∼3 mm × 3 mm. The purity and homogeneity are checked by electron microprobe analysis.

### High pressure experiments

Single crystals of MoSe_2_ were used for the high pressure IR, Raman, and resistivity measurements. Powders of MoSe_2_ were grounded from single crystals for the high pressure XRD measurements. Ruby spheres were used for determining pressure for all experiments. Neon was used as the pressure transmitting medium for the XRD and Raman experiments. Angle dispersive XRD experiments were performed at beamline 16-BMD of the Advanced Photon Source (APS), Argonne National Laboratory (ANL). The Rietveld fitting was performed using GSAS-EXPGUI package^58^. The Raman spectra were collected using a Renishaw inVia micro Raman system with a 514 nm laser excitation line. High-pressure IR measurements were conducted at beamline U2A of the National Synchrotron Light Source (NSLS), Brookhaven National Laboratory (BNL). A MoSe_2_ single crystal was sandwiched between the pressure transmitting medium (KBr) and one side of the culet. Infrared measurements were performed on a Bruker Vertex 80v FT-IR spectrometer coupled to a Hyperion-2000 microscope with a MCT mid-band detector. Fringes in raw IR data were removed by filtering high frequency terms after Fourier transformation. For temperature-dependent four-probe resistivity measurement, cubic BN was used as the insulating layer, and four electrodes were cut from Pt foils. The temperature-dependent sheet resistance of the sample was measured with Van der Paul geometry by cooling down to 10 K in a liquid helium cryostat. Pressures were measured at room temperature. More details are described in the supplementary information.

### *Ab-initio* calculations

The Vienna *ab-initio* Simulation Package^59^,^60^ was employed to optimize crystal structures and calculate electronic structures with the framework of local density approximation density functional theory^61^. The projector augmented wave^62^ pseudo-potential was used and the kinetic energy cutoff was fixed to 450 eV for all the calculations. For structural calculations in comparison with experiments, the unit cell volume is fixed while the cell parameters and atomic positions are fully relaxed. For band structure calculations, the lattice constants were fixed to be experimental values, and then the atomic positions are fully relaxed. HSE06 hybrid function^63^ was employed to improve the band structure calculations.

## Additional information

**How to cite this article:** Zhao, Z. *et al.* Pressure induced metallization with absence of structural transition in layered molybdenum diselenide. *Nat. Commun.* 6:7312 doi: 10.1038/ncomms8312 (2015).

## Supplementary Material

Supplementary Information Supplementary Figures 1-9, Supplementary Table 1, Supplementary Notes 1-6 and Supplementary References
