# Supplementary material for: Pressure induced metallization with absence of structural transition in layered molybdenum diselenide
Source: Nat Commun. 2015 Jun 19;6:7312. doi: 10.1038/ncomms8312 (PMC4557307; doi:10.1038/ncomms8312)
Supplement: Supplementary Information — Supplementary Figures 1-9, Supplementary Table 1, Supplementary Notes 1-6 and Supplementary References [file ncomms8312-s1.pdf]

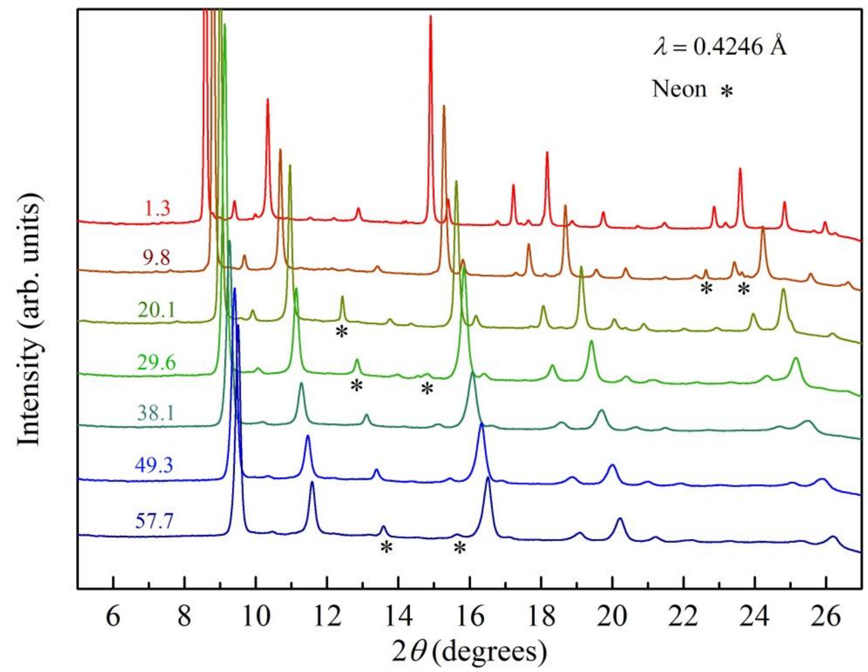

Supplementary Figure 1. Representative XRD patterns under pressure. Numbers represent pressures in unit of GPa. Asterisks indicate diffraction peaks from the pressure transmitting medium neon.

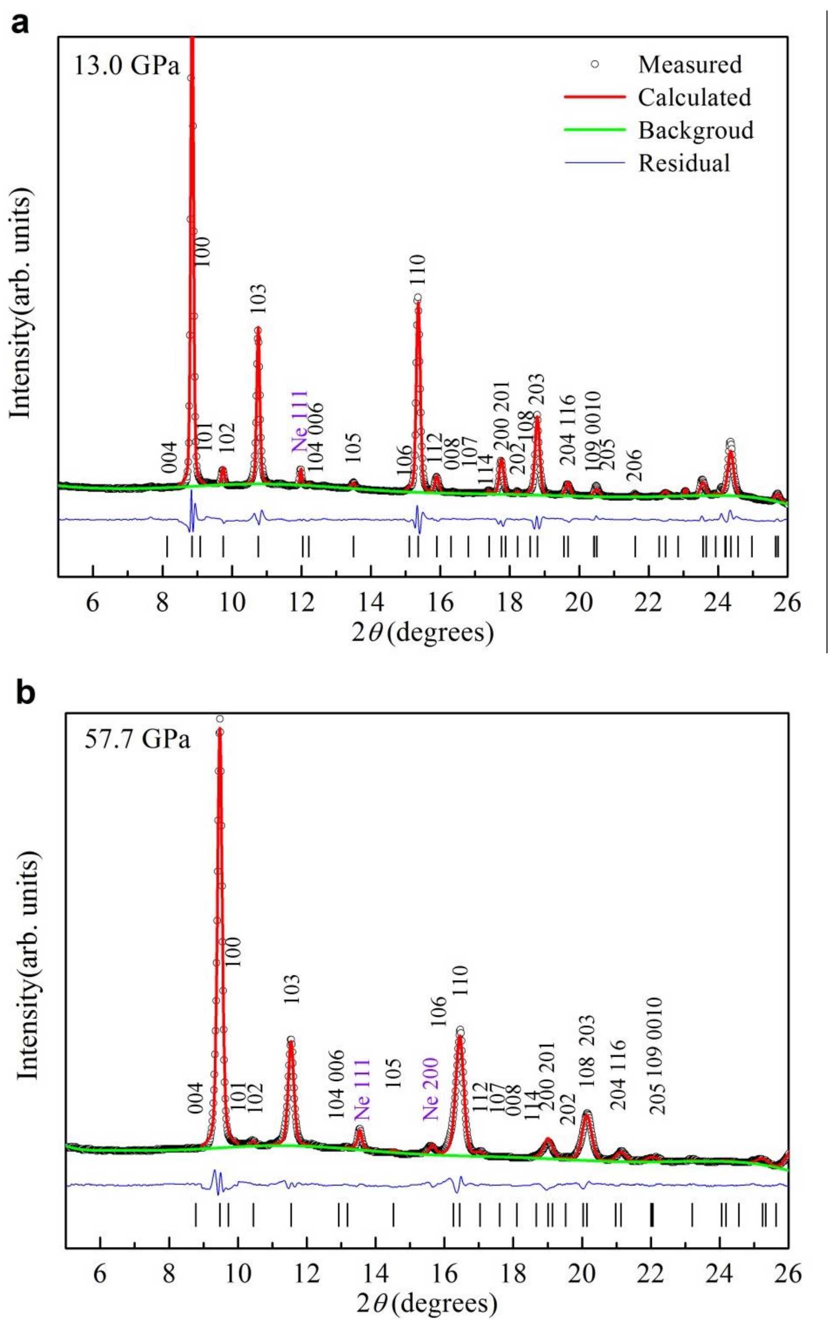

Supplementary Figure 2. Representative Rietveld refinement results for XRD data at (**a**) 13.0 GPa and (**b**) 57.7 GPa. The red lines and open circles represent the Rietveld fit and the measured counts respectively, and the blue lines give the residual intensities. The vertical bars indicate the predicted peak position of $MoSe_2$. Black labels show the diffraction peaks index of $MoSe_2$, and purple ones are from the pressure medium neon.

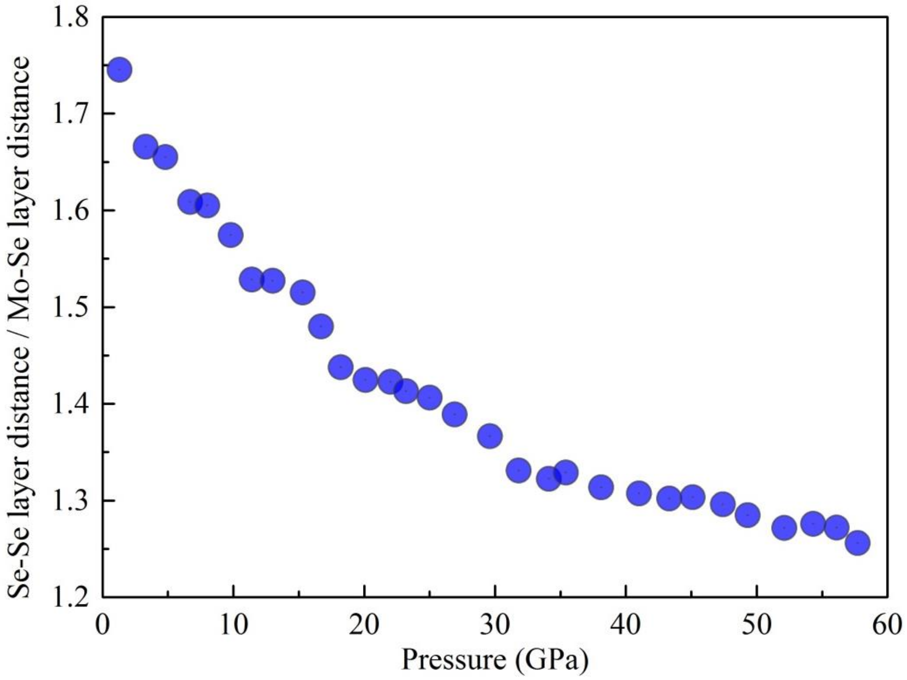

Supplementary Figure 3. Ratio of Se-Se layer distance to Mo-Se layer distance under pressure. This ratio is determined by the atomic positions from the Rietveld refinements.

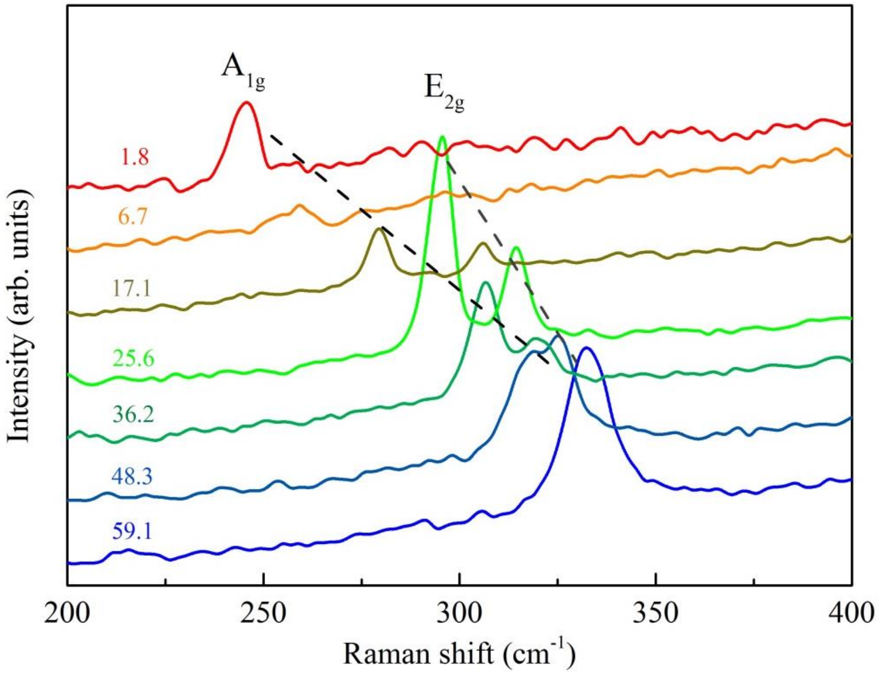

Supplementary Figure 4. Representative Raman spectra under pressure. Numbers represent pressures in unit of pressure.

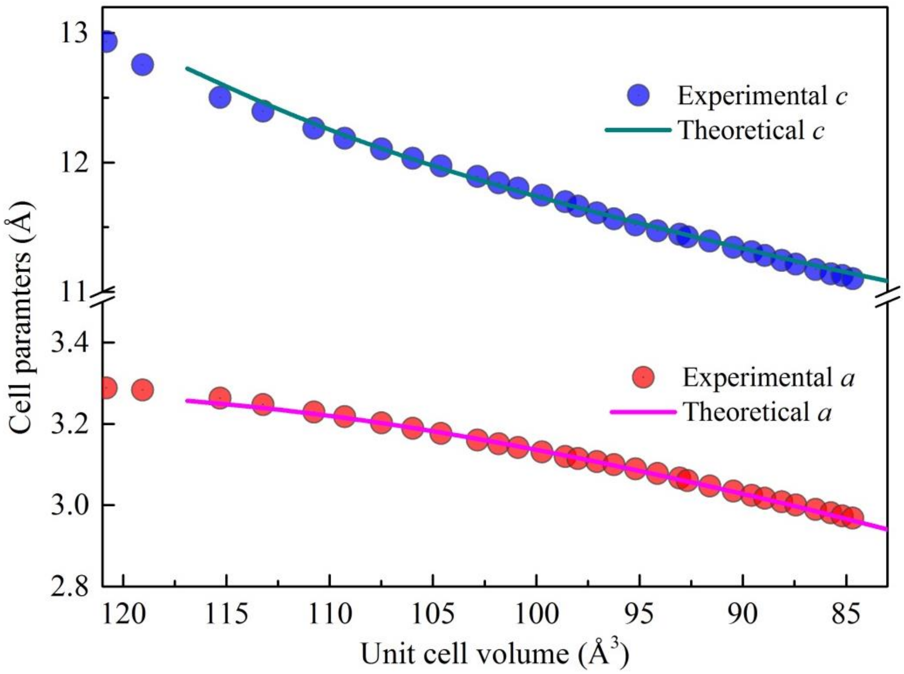

Supplementary Figure 5. Comparison of experimental and theoretical cell parameters at different unit cell volumes. Circles represent experimental data and lines are from calculations.

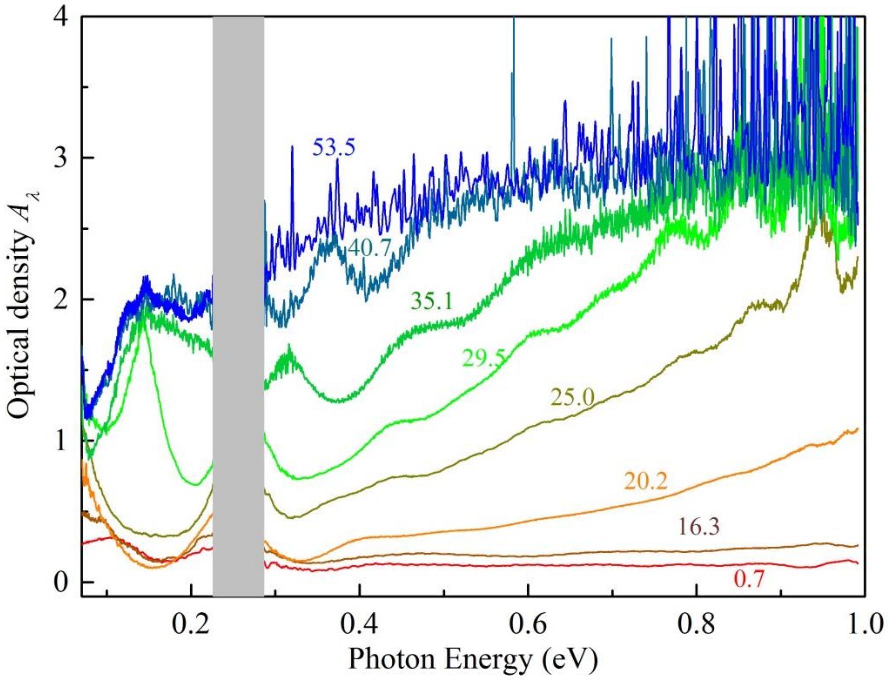

Supplementary Figure 6. Representative OD vs photon energy curves under pressure. The 0.23-0.28 eV region is obscured by diamond absorption. Numbers represent pressures in unit of GPa. The fluctuation of data (OD between 2 and 4) mainly results from the sharp decrease in transmittance under pressure, e.g. OD = 3 means transmittance = 0.001, which then makes the noise comparable to signal.

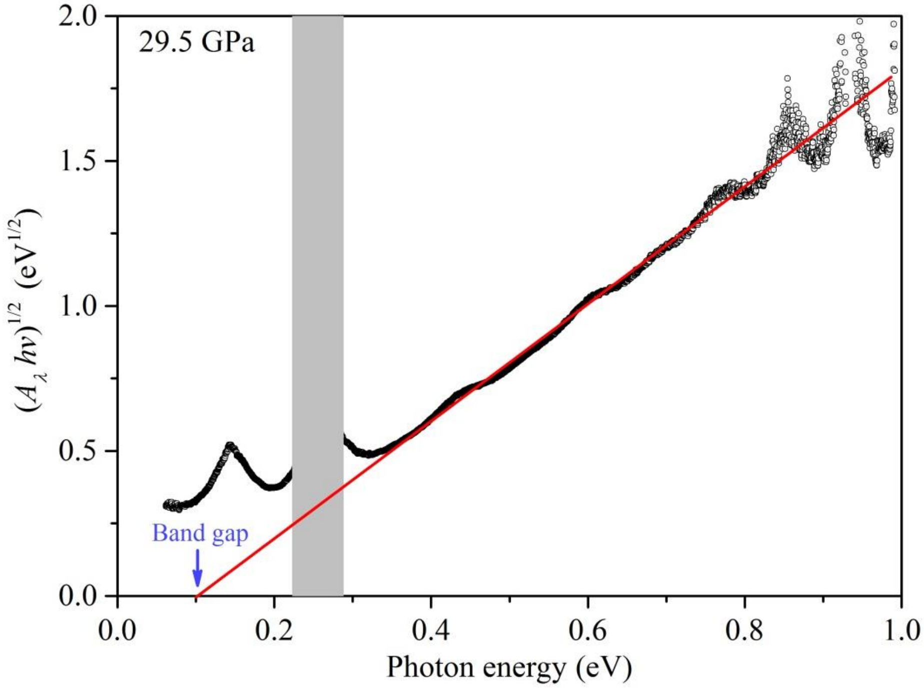

Supplementary Figure 7. Representative extrapolation for the indirect band gap at 29.5 GPa. The 0.23-0.28 eV region is obscured by diamond absorption.

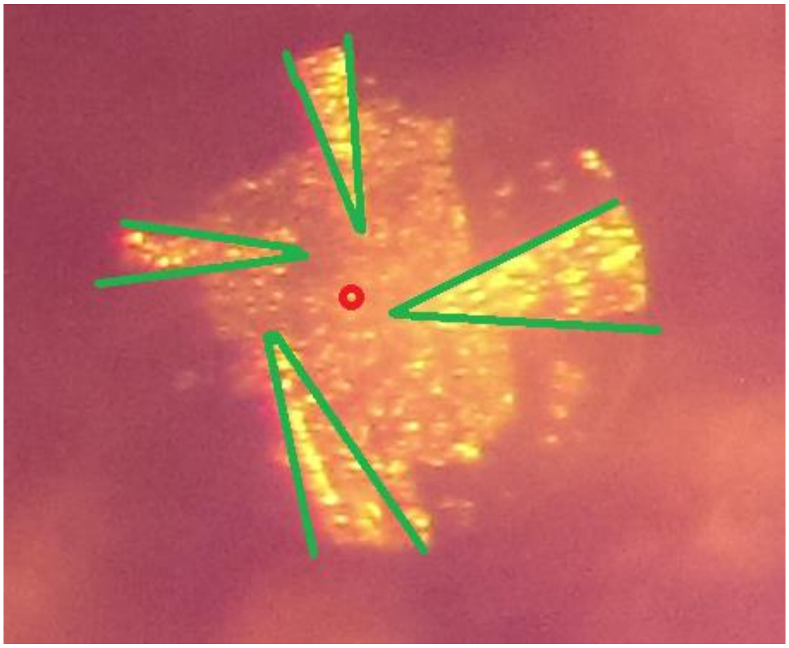

Supplementary Figure 8. Microphotography of $MoSe_2$ with four Pt electrodes attached to inside a DAC. The image is taken at ~ 30 GPa.

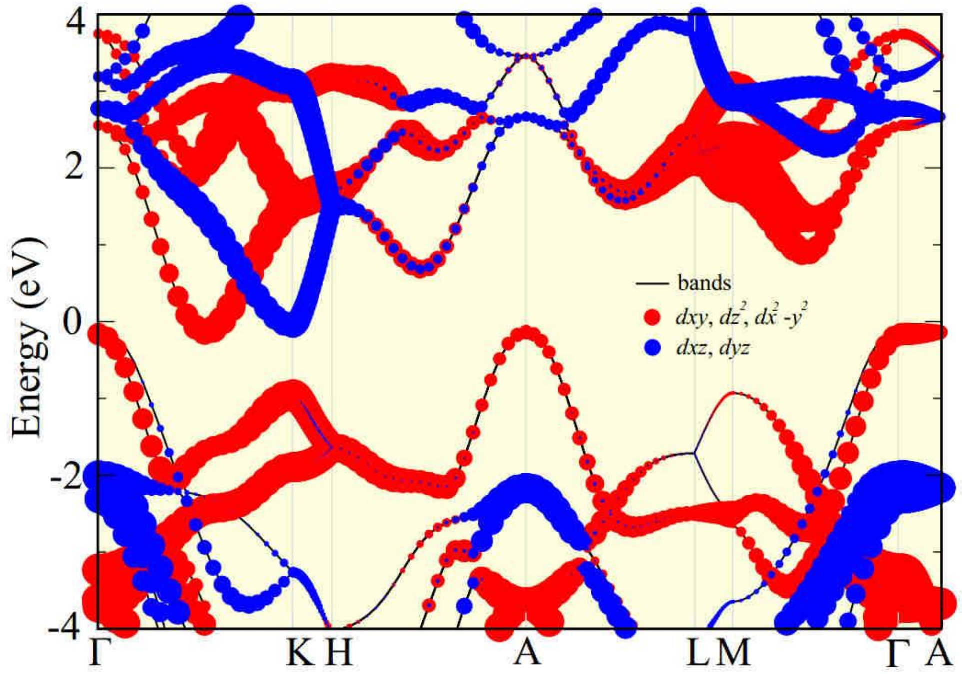

Supplementary Figure 9. Orbital information from band structure calculations at 41 GPa. The size of dot is proportional to the density of state of electrons.

Supplementary Table 1. Representative Rietveld refinement results from XRD data. Errors are given by GSAS-EXPGUI package.

Pressure (GPa)	Space group	Volume (Å^3)	Cell parameter *a* (Å)	*c* (Å)	Atom	Position parameter x	y	z
13	$P\,6_3/mmc$	104.6(1)	3.176(1)	11.974(2)	Mo	1/3	2/3	1/4
					Se	1/3	2/3	0.892(1)
31.8	$P\,6_3/mmc$	94.1(1)	3.078(1)	11.471(2)	Mo	1/3	2/3	1/4
					Se	1/3	2/3	0.900(1)
57.7	$P\,6_3/mmc$	84.7(1)	2.969(1)	11.099(2)	Mo	1/3	2/3	1/4
					Se	1/3	2/3	0.904(1)

Supplementary Note 1: X-ray diffraction measurements and analysis

Synchrotron X-ray diffraction (XRD) data was measured in beamline 16-BMD, Advanced Photon Source (APS), Argonne National Laboratory (ANL). The sample-detector distance was set at 304.32 mm with X-ray wavelength at 0.4246 Å. Neon served as the pressure transmitting medium. MAR345 image plate was used to collect the diffraction data. The 2D diffraction data with diffraction rings was then integrated into 1D diffraction patterns through Fit2D program. During compression, all diffraction peaks continuously shift to higher 2θ (smaller d-spacing) as seen from Supplementary Fig. 1. No new diffraction peak from $MoSe_2$ is seen throughout our measurements. Notice that diffraction peaks from neon (marked by asterisks) start to appear at 9.8 GPa. Decompression run shows that the shifts of all peaks are reversible.

We performed Rietveld refinement, a commonly used least-squares approach in solving powder XRD data, using the GSAS-EXPGUI package[1]. Representative Rietveld refinement results are shown in Supplementary Table.

In Supplementary Fig. 2, Rietveld refinement results for XRD data at 13.0 GPa and 57.7 GPa are shown. The red lines and open circles represent the Rietveld fit and the measured counts respectively, and the blue lines give the residual intensities. The vertical bars indicate the predicted peak position of $MoSe_2$. Black labels show the correspondent diffraction peaks index of $MoSe_2$, and purple ones are from the pressure medium neon. The $2H_c$-type structure can well fit all XRD patterns which supports the absence of structural transition.

The refined atomic positions then determine the ratio of Se-Mo layer distance to Se-Se layer distance. Supplementary Fig. 3 shows the ratio of Se-Mo layer distance to Se-Se layer distance, which drops fast at low pressure and but decreases much slower at high pressure. It indicates the gradual closure of the van der Waals (vdW) gap in between Se-Se layers.

Supplementary Note 2: Raman measurements

To probe the change of phonon modes of $MoSe_2$ under pressure, we measured its Raman spectra at high pressure. The spectra were collected using a Renishaw inVia micro Raman system with a 514 nm laser excitation line, at Extreme Environments Laboratory, Stanford University. Neon served as the pressure transmitting medium. Silicon's 520.4 cm^{-1} line was used for spectrum calibration before our measurements. To avoid overheating our sample, the laser intensity was set at 1 mW. The collection time was set at 60 s for each data point.

Representative Raman spectra during compression are shown in Supplementary Fig. 4. Two Raman modes A_{1g} and E_{2g} shift continuously under pressure. Each peak is fit by a lorentzian function using PEAKFIT. At above 57 GPa, these two peaks become so close that they could not separate by peak fitting. Notice that no new Raman mode is seen, which further supports the stability of the $2H_c$ structure. Decompression experiments show the shifts of these peaks are reversible.

Supplementary Note 3: Theoretical and experimental unit cells.

The Vienna *ab-initio* Simulation Package (VASP)[2,3] was employed to optimize crystal structures and calculate electronic structures with the framework of local density approximation density functional theory[4]. The projector augmented wave (PAW)[5] pseudo-potential was used, and the kinetic energy cutoff was fixed to 450 eV for all the calculations.

Supplementary Fig. 5 shows the comparison of theoretical and experimental cell parameters. The theoretical $2H_c$ unit cell information is obtained with fixed volume and fully relaxed lattices and atoms. The experimental data is from the Rietveld refinements, as shown in Supplementary Note 1. Circles represent experimental data and lines are from calculations. Good agreements are found in between the theoretical and experimental data. It needs to be pointed out that the largest discrepancy is at large volume (low pressure), where the calculated c is slightly larger than the experimental value, and the calculated a being smaller. This results from the well-known inadequacies of *ab-initio* calculations in describing the weak forces − in this case the vdW force (in between Se-Se layers).

Supplementary Note 4: Infrared measurements and analysis

High-pressure IR measurements were conducted in beamline U2A of the National Synchrotron Light Source (NSLS), Brookhaven National Laboratory (BNL). A $MoSe_2$ single crystal (thickness ~ 4 μm) was sandwiched between the pressure transmitting medium (KBr) and one side of the culet. Infrared microspectroscopy was performed on a Bruker Vertex 80v FT-IR spectrometer coupled to a Hyperion-2000 microscope with a MCT mid-band detector. Fringes in raw IR data were removed by filtering high frequency harmonic after Fourier transformation. Supplementary Fig. 6 shows Representative optical density (OD) vs photon energy curves under pressure. OD is defined as –log(T), T is transmittance. There is no sharp cut-offs in these curves, which supports the "indirect" feature in the electronic structure. The band gap closure is seen from gradual lifting of these curves.

For an indirect-band-gap semiconductor, the absorption coefficient is proportional to the square of the photon energy and band gap. Using this empirical model for semiconductors, we obtained the indirect-band-gap E_g via linear extrapolations of $(h\upsilon A_\lambda)^{1/2}$. A representative fitting at 29.5 GPa is shown as result Supplementary Fig. 7.

Supplementary Note 5: Electrical resistivity measurements

For temperature-dependent four-probe resistivity measurement, cubic BN was used as the insulating layer and pressure transmitting medium. A single crystal of $MoSe_2$ with suitable size was chosen for measurement. Ruby was used as the pressure calibrant. The four electrodes with sharp heads were cut from Pt foils, see Supplementary Fig. 8. The Van der Pauw geometry of these four electrodes is outlined by green lines. The position of ruby is marked by red circle. The temperature-dependent sheet resistance of the sample was measured by cooling down to 10 K in a liquid helium cryostat after changing each pressure at room temperature. The difference in pressures before and after the cooling and warming cycle is typically ~ 5 %.

Supplementary Note 6: Orbital details in band structure

HSE06 hybrid function[6] was employed for calculations. The **k**-points mesh is taken as 12 × 12 × 10 for all bulk self-consistent calculations. The conduction bands and valence bands near the E_F show large movements. At ambient pressure, the *dxz* and *dyz* dominated conduction bands are further away from the Fermi level than the *dxy* and $dx^2 - y^2$ dominated bands. Interestingly, two band minima are observed at high pressure. For example, at 41 GPa as seen in Supplementary Fig. 9, one *dxz* and *dyz* dominated conduction band quickly goes down at K point to form two conduction band minimum. This is because that *dxz* and *dyz* orbitals gain more overlap with Se *p* orbitals to widen the band dispersion than *dxy* and $dx^2–y^2$ within the $2H_c$ structure under pressure. The conduction band minimums may play a role in determining the transport properties.

Supplementary references:

1. Toby, B. H. EXPGUI, a graphical user interface for GSAS. *J. Appl. Cryst.* **34,** 210–213 (2001).

2. Kresse, G. & Joubert, D. From ultrasoft pseudopotentials to the projector augmented-wave method. *Phys. Rev. B* **59,** 1758 (1999).

3. Kresse, G. & Hafner, J. Ab initio molecular dynamics for liquid metals. *Phys. Rev. B* **47,** 558 (1993).

4. Hohenberg, P. & Kohn, W. Inhomogeneous electron gas. *Phys. Rev.* **155,** 864 (1964).

5. Blöchl, P. E. Projector augmented-wave method. *Phys. Rev. B* **50,** 953 (1994).

6. Heyd, J., Scuseria, G. E. & Ernzerhof, M. Hybrid functionals based on a screened Coulomb potential. *J. Chem. Phys* **124,** 8207 (2006).
